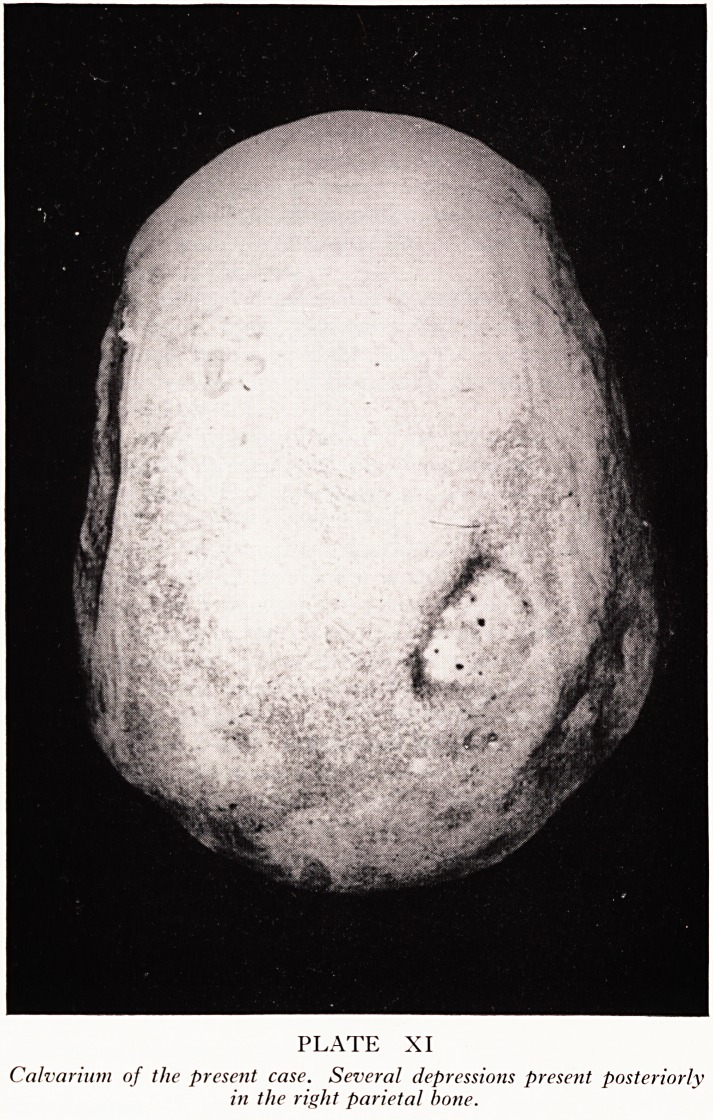# Intestinal Reticulosarcoma in Idiopathic Steatorrhoea

**Published:** 1964-04

**Authors:** 


### INTESTINAL RETICULOSARCOMA IN IDIOPATHIC STEATORRHOEA

*Dr. Naish:* I first saw this man in 1955 and he was then in his middle forties. At that time he had a small pneumothorax, he was very thin and gave a history of loose stools for many years. As well as investigating his pneumothorax we investigated his loose stools and I thought that he was suffering from idiopathic steatorrhoea. I thought he had probably had this for a number of years. My diagnosis was confirmed only by finding excess excretion of fat in the stools. There were no major deficiencies as a result of this. He was put on a low fat, high protein diet with vitamin supplements, and did fairly well.

In 1958 he was again admitted to hospital with symptoms of reflux oesophagitis which we thought were secondary to hiatus hernia. After treatment for the oesophagitis his diarrhoeal condition worsened and he slowly lost weight over the next 2 years, so in June 1960 he was readmitted for us to reconsider the whole question of diagnosis, and at this time we were able to make a fuller study of the man. He had a flat glucose tolerance curve, xylose absorption was so poor that he only excreted 1·9 g in his urine over 5 hours after an oral dose of 25 g. His serum calcium was low, and the fact that he had trypsin in his stools suggested that he did not have chronic pancreatitis. His proteins were lowered, particularly the albumin, and we were able to do an intraluminal jejunal biopsy, and this specimen of jejunal mucosa showed fairly typical changes of idiopathic steatorrhoea, namely blunting of the villous structure. He was at that time started on a gluten-free diet and the response was disappointing. He did not gain more than a few pounds of weight although his diarrhoea and his morale improved.

I saw him once or twice in Outpatients. He was in difficult social circumstances, having been a widower for several years and he was trying to educate a couple of daughters. He did a lot of housework as well as his ordinary work, and was a man of considerable fortitude.

Then one day I received a telephone message from Professor Milnes Walker's house surgeon at the B.R.I., asking what treatment he had been having, because he had been admitted to the B.R.I. as an acute abdominal emergency, either perforation or intestinal obstruction.

About six months before this he had been put on treatment with cortico-steroids in addition to the gluten-free diet in order to improve his nutrition, so my first thought on receiving this message was that the therapy had caused a peptic ulcer to perforate or something of that sort. I told the house surgeon details of the treatment and they went ahead and operated.

*Dr. Read:* At operation a tumour was found in the ileum which had perforated, and the tumour and the surrounding piece of bowel was resected. I was invited to see him after he had had this resection and I said, wrongly as it turned out, that these two conditions were quite unrelated. I agreed that he had idiopathic steatorrhoea or adult coeliac disease, and that he now had malignant tumour, as it turned out a reticulosis of the bowel. I thought that these were two separate things. I did not make one mistake, however, and say that the whole picture was due to a reticulosis. This was clearly a localized tumour, and although one sees involvement of the small bowel in reticulosis quite often, steatorrhoea is rather rare. In a large series analysed by Rosenberg in 1961, of 1,200 cases of reticulosis only 138 had small bowel involvement and there was no evidence that any of them had steatorrhoea. It is a rare event but it would have been a way of linking these two diseases. However, we did not link them together like that, we merely said that they were unconnected.

He was diagnosed as having secondaries from the reticulosis on the scalp. He also had at that stage, and this is very important, exfoliative dermatitis affecting most of the body, which most people thought was associated with the reticulosis. Once his abdominal wound was well healed he went to B.G.H. for radiotherapy and had 2,500 R to his scalp to get rid of the tumours on his head. They melted away, and this further supported their being secondary reticulosis. These were never biopsied and had been diagnosed by his doctor as sebaceous cysts and he was awaiting an appointment at the casualty theatre for removal of these cysts when in fact he perforated!

The story then becomes difficult and I cannot join it up without referring to one or two other patients.

We had at that time in the wards a lady who had had idiopathic steatorrhoea for two years and who was handed on to me by Dr. Sutton. She started to go downhill. She complained of abdominal pain, ran a pyrexia, and when we got her into the ward it was obvious that she had a mass in her abdomen. When Mr. Horton opened up this lady with idiopathic steatorrhoea, he found she had a tumour. Professor Hewer saw the tumour and thought it looked much the same as the one from the present case. Well now, one case is an anecdote, two cases are a paper! So I telephoned Dr. Naish and told him I considered this important and suggested that we should write these cases up, and he replied that in the dim distant past he had had a third case and he would send me the notes, which he did.

At about this time I met a Dr. J. Bolten who told me of a patient of his who had had idiopathic steatorrhoea for nine years and who had been doing very well on a gluten-free diet but who had recently begun to go downhill, with abdominal pain and a skin rash. It was arranged that I should see this patient, and by a peculiar twist of fate this gentleman suddenly became very ill and was admitted as an emergency complaining of abdominal pain. This man had a scaly eruption over most of his body which was itchy. He also had idiopathic steatorrhoea and had perforated.

Now to return to this evening's case; at this time I met the patient's daughter who told me that he was very ill and was going downhill. As a result we got the man into hospital. He became wasted and miserable, although he did not have much trouble from his intestinal complaint he developed two interesting extra complications: a frozen shoulder, i.e. a painful right shoulder with severe limitation of movement which needed a lot of physiotherapy and made him depressed, and secondly pyramidal tract signs on the left side. Of course, we remembered the story of tumours on the scalp and wondered whether there had been invasion of the brain, or whether the 2,500 R had damaged the brain, or whether it was a simple cerebral thrombosis. We noted the signs but did not know what they meant. From then on he went slowly downhill. We did not repeat the intestinal tests, there was no point, and eventually he died. As soon as he died dilute formalin was put into the alimentary tract to preserve it.

*Professor Hewer:* You thought that the pyramidal signs might conceivably be due to the tumour in the scalp invading the brain? In which case presumably there would have been some changes in the skull in life?

*Dr. Gordon:* The initial picture in January 1961 shows irregular translucent areas in the skull—we did not really know what these were, but they looked very like the changes that you get in reticulosis of the skull bones. We thought we could make out that it seemed to be affecting the outer table, but leaving the inner table more or less intact. From that we made the suggestion that these could have arisen perhaps from a lesion in the scalp which was pressing on the skull and eroding the bone and that was as far as we got in the way of saying what these might be. X-rays taken in October 1961 show that the translucent areas have become smaller and are more sharply defined. What that means I am not sure, but it appeared that things had improved following the radiotherapy.

*Dr. Sanerkin:* Did you consider the possibility that the neurological signs and symptoms might be due to cerebral demyelination which sometimes complicates cases of reticulosis?

*Dr. Read:* This was another possibility. I am afraid I had forgotten about the second course of X-rays. Dr. Gough reminded me of it. We did think of demyelination itself—direct radiation insult, and perhaps local invasion.

*Dr. Sanerkin:* Was there any evidence of osteomalacia?

*Dr. McGowan:* Not biochemically; his plasma alkaline phosphatase and phosphate levels were normal throughout, and although his plasma level was low on occasions, this could be accounted for by co-existing low plasma protein levels.

*Dr. Sanerkin:* Any evidence of anaemia?

*Dr. Read:* He had no anaemia. Dr. Naish had been treating him with folic acid for some time so that is not surprising.

*Dr. McGowan:* His plasma protein concentration varied, but was low for most of the time, especially the albumin, as one would expect in a case of malabsorption. His xylose absorption was consistently low, less than 15 per cent of a 25 g dose being excreted in the urine over 5 hours. A glucose tolerance test was not performed.

*Dr. Read:* These are tests of upper alimentary function simply because this is the first part of the alimentary tract that these substances meet and most are simple substances and therefore easily absorbed. If there is an abnormality of xylose or glucose absorption this tells you that there is involvement of the upper alimentary tract. The type of lesion with severe jejunal involvement is adult coeliac disease or coeliac disease in children, so it is very helpful.

*Dr. Naish:* In the previous man you mentioned I believe you related the presence of the rash to reticulosis; I am not happy about this myself. I have seen a rash which is like that in other patients with steatorrhoea in a terminal stage and at post mortem they have not had a tumour. I believe it is some sort of nutritional rash. A crazy-paving pattern of streaking of the skin seems to me a typical finding in the last stages of severe malabsorption. As I say, I have seen this a number of times in patients with steathorrhoea who do not have reticulosis.

*Dr. Read:* Did they have pruritus?

*Dr. Naish:* I don't remember. In this patient, there was an erythematous rash which was almost macular. But that is different. It is the crazy-paving rash which I think is nutritional.

*Dr. Sanerkin:* Did this man's rash persist to the end?

*Dr. Read:* No, it did not. It disappeared when the reticulosis disappeared, i.e. after the X-ray therapy to the scalp. I agree with Dr. Naish that rashes are common in idiopathic steatorrhoea. We do not know why they appear but I think the fact that both these men developed widespread skin rashes, both having a reticulosis, is suggestive particularly as neither had had a skin rash before. I think they were connected, in fact that reticulosis does cause skin rashes of this type.

*Professor Hewer:* Obviously the whole thing is very complex. There is a significantly high proportion of eczematous and psoriasiform rashes in people with steatorrhoea. I believe we have a dermatologist here tonight?

*Dr. Walshe:* Any red skin could be called an erythrodermia. If the skin itself is involved in the reticulosis it may develop an exfoliative eczema. It may well be infiltrated or develop tumours. Pruritus is very common in reticuloses and often there is nothing to see but scratch marks. One thing that occurred to me here is that the low serum calcium might have had an effect on the skin. Other lesions described in association with steatorrhoea include eczema, ichthyosis, and pigmentation, and changes related to vitamin deficiencies.

*Professor Neale:* Can you tell us something about the chemical substances in the urine in steatorrhoea? Was the urinary indole test performed in this case?

*Dr. Read:* Dr. McGowan will tell us that, but increased bacterial activity in the gut may cause increased indole excretion. I think this is well accepted as an indicator of abnormal breakdown of protein.

*Dr. McGowan:* So far as I know, we were not asked to test for an excess either of indole in the urine or of coproporphyrin in the faeces. Both are commonly raised in conditions of malabsorption. They can both be restored to normal by suitable antibiotics, so I agree with Dr. Read that they are probably due to bacterial activity in the intestines.

*Dr. Sanerkin:* At necropsy, the body was grossly wasted. There was an old operation scar on the abdomen, dating from the removal of the intestinal tumour. I could not detect any rash in the skin. There were no cutaneous tumours, certainly none in the scalp. I will come to that later.

He had brown atrophy of the heart, which weighed 235 gm. There were two healed cardiac infarcts with dense fibrous replacement of the myocardium. One was situated posteriorly above and behind the posterior papillary muscle, the other anteriorly high up in the ventricular septal muscle. There was an old organized occlusion of the right coronary artery: the anterior descending coronary artery was severely atheromatous and stenosed but not occluded. The immediate cause of death was bronchopneumonia.

The alimentary tract was quite satisfactorily preserved because the physicians had injected formalin into the abdominal cavity and into the stomach and gut soon after death. I did not find any hiatus hernia and there was no oesophagitis. The mucosa of the stomach was flattened out and smooth through gastric atrophy, the presence of which was confirmed by histology.

I shall describe the main, or intestinal, lesions in two groups, dealing first with the idiopathic steatorrhoea and then with the reticulosarcoma. In the duodenum and jejunum the villous pattern of the mucosa was destroyed because of the villous atrophy (Plate VII) which is found in association with idiopathic steatorrhoea. The entire small intestine had a distinct reddish-brown colour due to pigmentation of the muscular coat. This pigment is a lipochrome, lipofuscin, better known as "wear and tear pigment"; it is brown in colour, stains positively with P.A.S. and sudan black, and is often acid-fast with Ziehl-Nielsen. Such pigmentation is a common finding in idiopathic steatorrhoea although it may also be seen in other alimentary disorders, including Whipple's disease and jejunal diverticulosis, and in some cases of hepatic cirrhosis as well.

I found no evidence of the two common complications of idiopathic steatorrhoea. There was nothing to suggest anaemia and the femoral marrow was fatty, not red and hyperplastic as one might expect in presence of macrocytic or other deficiency anaemia. There was no osteomalacia. The bones were easier to cut than normally, but this was due to osteoporosis. The parathyroids were, however, obviously hyperplastic, being two to three times larger than those seen in a case of chronic nephritis with osteitis fibrosa. This parathyroid hyperplasia must reflect the disturbed calcium/phosphate metabolism.

With regard to the reticulosarcoma, the specimen removed at operation is shown in Plates VIII and IX. The tumour is fungating through the mucosa, and tumorous lymph nodes are present in the adjoining mesentery. At necropsy, the scar of the surgical resection was found about 110 cm below the duodeno-jejunal junction and was quite free from recurrent tumour. Two fresh tumours were, however, present; one about 15 cm below the duodeno-jejunal junction, the other about 50 cm further down. They were small button-shaped masses situated in the submucosa, ulcerating the overlying mucosa. Histologically, sections from the surgical and necropsy tumours showed a typical reticulosarcoma (Plate X) growing in diffuse sheets of fairly uniform reticulum cells in a fairly rich stroma of reticulin fibrils. Occasional tumour giant cells were found and mitoses were frequent. Similar tumour was seen in several enlarged lymph nodes in the mesentery. At all other sites the lymph nodes were quite small and free from tumour. There was no histological evidence of hepatic or splenic involvement,

PLATE VII

*Jejunal mucosa at necropsy showing total villous atrophy due to idiopathic steatorrhoea. Haematoxylin and Eosin.* ×140.

PLATE VIII

*Surgical specimen showing resected small intestine. Fungating tumour ulcarting through the mucosal surface.*

PLATE IX
*Section through one of the jejunal tumours found at necropsy, showing the submucosal tumour with ulceration of the overlying mucosa. Villous atrophy of jejunal mucosa also clearly shown. Haematoxylin and Eosin.* ×4.

PLATE X
*Histological appearance of jejunal tumour, showing sheets of reticulum cells. Haematoxylin and Eosin.* ×620.

PLATE XI

*Calvarium of the present case. Several depressions present posteriorly in the right parietal bone.*

although these organs readily become involved with systematization of lymphoid tumours. The spleen was indeed rather atrophic, weighing only 95 gm.

I examined the scalp very thoroughly but found no trace of tumour either macroscopically or histologically. The scalp hairs were generally sparse, but there were several sharply circumscribed bald patches. Histological examination of these patches showed only the results of irradiation, with disappearance or atrophy of the pilosebaceous units. The dermis and subcutis at this site were expanded by dense fibrous tissue. Tumour may well have been present here, especially in view of the clinical history. We have heard of a mass which melted away on irradiation. I can think of no benign lesion which would disappear without trace, certainly not a sebaceous cyst. Dr. Tudway told me, "If you had seen this patient, as I did, you would have had no doubt that this was a radiosensitive malignant tumour which disappeared with treatment". One can only assume that it was indeed a deposit of reticulosarcoma. It is a pity that no biopsy was taken from it before irradiation. I remember another case with idiopathic steatorrhoea and intestinal reticulosarcoma, also a patient of Dr. Read, who had mesenteric lymph node involvement but no other tumour except for a solitary nodule of reticulosarcoma in the lung.

In the skull I found several depressions posteriorly in the right parietal bone (Plate XI). One may presume, again, that these depressions may have been caused by tumour which disappeared following radiotherapy. I cannot, however, exclude the possibility that these depressions might be natural phenomena of parietal thinning of the type which Dr. Lloyd collects.

On slicing the brain after fixation I noticed multiple tiny discrete lesions, greyish and not softened, scattered throughout the white matter, occasionally encroaching upon the cortical grey matter. A few similar foci were also present in the brain stem. Considerable difficulty was experienced in the interpretation of these lesions. They are unlikely to have been micro-infarcts, which are usually yellowish and soft or cystic. Direct damage by the irradiation is also highly improbable. Dr. Tudway told me that higher doses of irradiation are given without producing cerebral damage. One must give about 4,000 R to produce brain damage; this man had only about half that dose. Another possibility is that these lesions were once occupied by reticulosarcoma deposits which disappeared after radiotherapy. The widespread distribution of the lesions makes this improbable. The most reasonable explanation is that of disseminated demyelination which sometimes occurs as a non-neoplastic complication of primary lymphoid tumours. I have shown the sections to Dr. Urich and he has agreed to accept them as an example of such disseminated demyelination.

To sum up, then, this case shows a sequence of unusual complications: intestinal reticulosarcoma complicating idiopathic steatorrhoea and multifocal leuco-encephalopathy complicating the reticulosarcoma.

*Question:* Was the second lot of radiotherapy given *after* the appearance of neurological signs?

*Dr. Sanerkin:* Yes. Irradiation was repeated because it was thought that the neurological signs might be due to tumour deposits in the brain. This further eliminates radiotherapy as a cause of the cerebral lesions.

*Question:* Did you examine the spinal cord?

*Dr. Sanerkin:* No. I did not slice the brain at necropsy but kept it for fixation before full examination. I was not certain what to expect in the brain. What I eventually saw came as a complete surprise. If I had thought of multifocal leucoencephalopathy at the time of the necropsy I would certainly have taken out the cord also.

*Professor Hewer:* Perhaps Dr. Lloyd would like to tell us what he thinks about the changes in the skull?

*Dr. Lloyd:* I cannot accept this case of Dr. Sanerkin's as being the naturally occurring condition of symmetrical thinning of the parietal bones, but there are other forms of atrophy of the skull, of which this may be an example. Symmetrical parietal thinning occurs in a very characteristic situation and is a form of senile atrophy, probably. We do not know the cause of it. It only occurs in elderly people and affects the outer table and diploe and not the inner table. The only naturally occurring condition which can be confused with symmetrical parietal thinning is a congenital condition in which you get dilatation of the parietal foramina, but in those cases you will find the holes are in a different place.

*Professor Hewer:* Do we know for certain that the concavities of the skull are actually where the tumours of the scalp were?

*Dr. Sanerkin:* I do know that the depressions in the skull were underlying the scars in the scalp which were unmistakeable, big depressed thick white areas.

*Dr. Read:* I think the main thing in this case is that here is an example of idiopathic steatorrhoea complicated by a reticulosarcoma. Now we are all familiar with epithelial pre-malignant conditions such as chronic gastritis, chronic gastric ulcer, chronic ulcerative colitis, leading to carcinoma, but surely the occurrence of a reticulosis in an area with a disorganized mucosal surface is unusual.

*Dr. Cornes:* In 1935 Manson Bahr and Professor Pulverstaft published a series of patients who had steatorrhoea associated with reticulum-cell sarcomas of the small intestine. Their patients had had steatorrhoea from a few weeks to one and a half or two years' duration at the most. Some patients may have symptoms from lymphoid tumours of the gut for five or six years before they are seen. They concluded that the steatorrhoea was secondary to these lymphoid tumours, and not the other way round.

In the present case the patient had steatorrhoea for 15 years before a lymphoid tumour was found, and clearly in this case the lymphoid tumour is secondary to the steatorrhoea.

In investigating a series of 281 patients with malignant tumours of lymphoid tissue I found three patients with definite steatorrhoea. In the first two cases the patients had had steatorrhoea for only a short time. The third case, a woman aged 28 years with a reticulum-cell sarcoma of the small intestine, was known to have had steatorrhoea since the age of 3 years.

There does appear to be some association between epithelial disorders and lymphoid tumours of the gut.

*Professor Neale:* Could I ask how often you find lymphosarcoma or reticulosarcoma with any other physical or pathological findings as a primary condition. How far is it statistically fortuitous?

*Dr. Cornes:* I don't know. However, Sleisenger, Almy and Barr in New York discovered several patients with malignant tumours of lymphoid tissue in the gut wall who were shown to have steatorrhoea although steatorrhoea had not been diagnosed clinically.

*Dr. Read:* In that paper, Dr. Cornes, they had no evidence to prove their cases were in fact steatorrhoea.

*Professor Hewer:* It might be worth considering whether there are any abnormal substances formed in the gut, in subjects with steatorrhoea, that could, on absorption through the mucosa, be capable of initiating a malignant change in the lymphoid tissue. Do we know anything about the abnormal substances formed in the gut in steatorrhoea other than failure to digest the fats?

*Dr. Read:* I should have thought perhaps the very fact that the submucosa of these bowels was stuffed full of lymphocytes and inflammatory cells might be of some importance, and that the lymphoid tissue being spread out and not arranged in patches as it is in normal bowel may be of some significance.

*Dr. Cornes:* In the experimental animal there is an increase of lymphoid tissue in the intestinal wall during fat absorption. Examination of the small intestine from obese patients does show prominent lymphoid tissue compared with similar specimens from poorly nourished patients.

*Dr. Read:* We may think that this is a new disease, but Dr. Gough went through the literature very carefully and picked out twenty-nine cases of intestinal reticulosis where there had been steatorrhoea. He looked at them very carefully and reckoned that about sixteen of them had reasonable evidence that they were idiopathic steatorrhoea or coeliac disease or tropical sprue, three conditions in which the mucosa is abnormal and indistinguishable. Those of course were the days before intestinal biopsy and there was not very much evidence on which to base a confident diagnosis. At least half of these cases do seem to be this peculiar combination which we have brought to the surface again.

One other point which I think is important; I have since been asked to attend one other pathological conference where precisely the same thing has been demonstrated except that in this case it was Hodgkin's disease. It seems to show that the reticulosis can develop along any sort of path. I think it is pure coincidence that we have three reticulum-cell sarcomas. There seems to be some basic defect of lymphoid tissue developing in the alimentary tract, very rarely outside. It seems to start in the abnormal gut and this is the only case so far in which we have seen evidence of dissemination outside.

## Figures and Tables

**PLATE VII f1:**
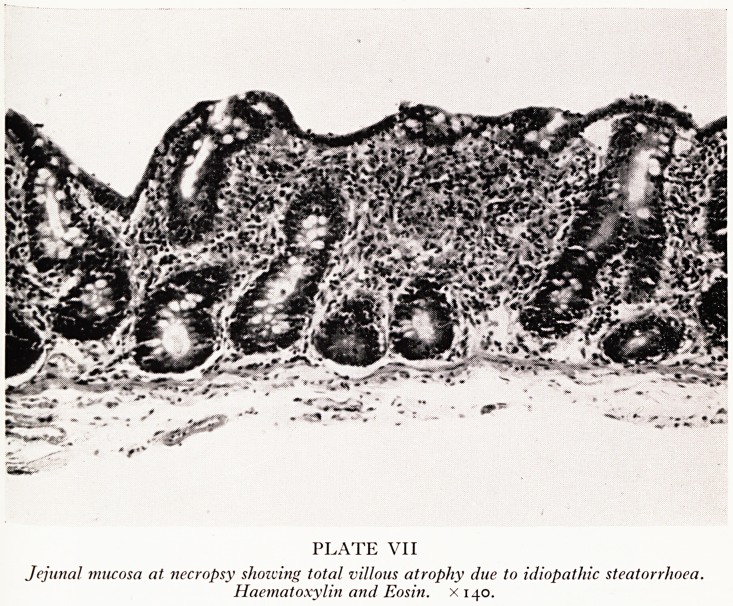


**PLATE VIII f2:**
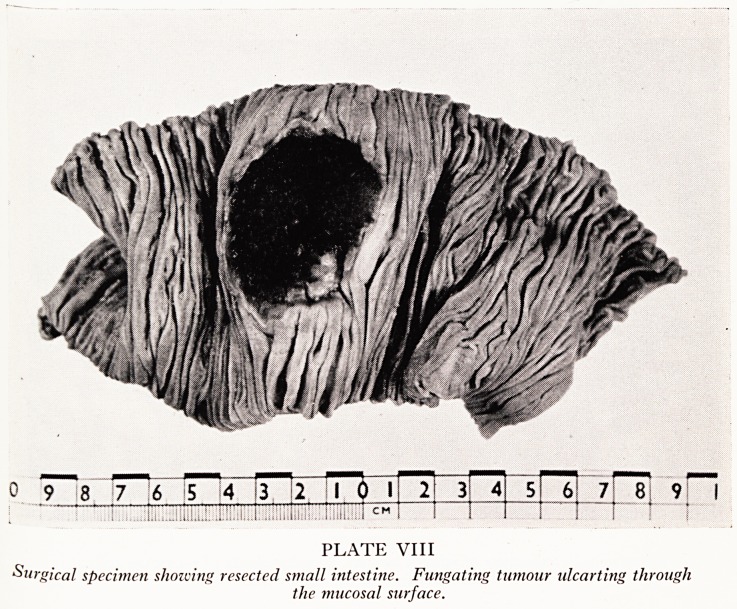


**PLATE IX f3:**
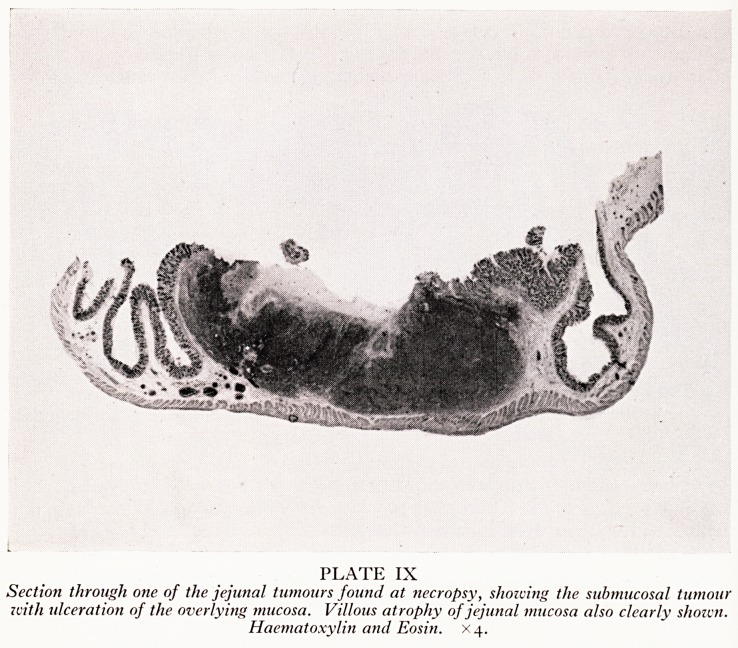


**PLATE X f4:**
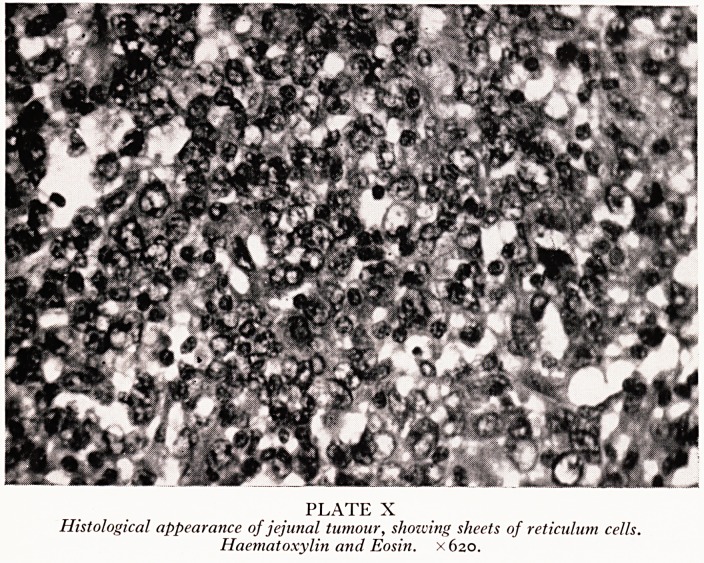


**PLATE XI f5:**